# Care of the Critically Ill Emergency Department Patient with Acute Kidney Injury

**DOI:** 10.1155/2012/760623

**Published:** 2011-11-24

**Authors:** Jennifer Joslin, Marlies Ostermann

**Affiliations:** Department of Critical Care, King's Health Partners, Guy's & St Thomas' Foundation Trust, King's College London, London SE1 7EH, UK

## Abstract

*Introduction.* Acute Kidney Injury (AKI) is common and associated with significant mortality and complications. Exact data on the epidemiology of AKI in the Emergency Department (ED) are sparse. This review aims to summarise the key principles for managing AKI patients in the ED. *Principal Findings*. Timely resuscitation, goal-directed correction of fluid depletion and hypotension, and appropriate management of the underlying illness are essential in preventing or limiting AKI. There is no specific curative therapy for AKI. Key principles of secondary prevention are identification of patients with early AKI, discontinuation of nephrotoxic medication where possible, attention to fluid resuscitation, and awareness of the risks of contrast-induced nephropathy. In patients with advanced AKI, arrangements for renal replacement therapy need to be made before the onset of life-threatening uraemic complications. *Conclusions*. Research and guidelines regarding AKI in the ED are lacking and AKI practice from critical care departments should be adopted.

## 1. Introduction

Acute kidney injury (AKI) is common in hospitalised patients, especially in those who are critically ill [1, 2]. The Kidney Disease Improving Global Outcomes (KDIGO) working group estimates a worldwide AKI prevalence of ~2100 people per million population [3]. 

There is increasing recognition that even a minor acute reduction in renal function is independently associated with a poor outcome, including higher risk of complications, longer stay in hospital, high mortality, and increased risk of needing long-term dialysis [4–9]. Although there is no curative therapy for AKI, research over the past decade has identified various means to prevent or limit AKI. Unfortunately, these are not widely known and are variably practised worldwide resulting in lost opportunities to improve the care and outcomes of patients with AKI. Importantly, there is no unifying approach to the care of these patients. There is a worldwide need to recognise patients at risk of AKI, to intervene early, and to circumvent the need for renal replacement therapy (RRT). 

Although a large proportion of patients presenting to the Emergency Department (ED) either have AKI or develop AKI later in hospital, the literature regarding epidemiology and management of such patients in the ED setting is lacking. In the UK, the recent National Confidential Enquiry into Patient Outcomes and Death (NCEPOD) report found that AKI is often underrecognised and poorly managed. The report made a strong call for better education and training [10]. To our best knowledge, neither the College of Emergency Medicine (UK), American College of Emergency Physicians, Australasian College for Emergency Medicine, nor Canadian Association of Emergency Physicians have published guidelines on management of AKI in the ED. This review aims to summarise existing knowledge about the management of AKI with particular emphasis on specific patient groups in the ED setting.

## 2. Definition of AKI

The literature contains >50 definitions for AKI which has posed significant problems for comparative epidemiology and clinical research. In the last 10 years, efforts have been made to standardise the definition of AKI. In 2004, the Risk-Injury-Failure-Loss-Endstage (RIFLE) criteria were published, followed by the revised AKI Network (AKIN) classification in 2007 [11, 12] (Table 1). Both definitions will soon be superceded by the KDIGO criteria which define AKI by any of the following:

- increase in serum creatinine by ≥0.3 mg/dL (>26.5 *μ*mol/L) within 48 hours;
- increase in serum creatinine to ≥1.5 times baseline, which is known or presumed to have occurred within prior 7 days; or
- urine volume <0.5 mL/kg/h for 6 hours.

The scale of the AKI burden in ED is not known. However, the key issue is that even relatively minor acute changes in serum creatinine independent of underlying aetiology represent significant deterioration of renal function and are associated with poor outcome [4].

## 3. Pathophysiology of AKI

There is increasing evidence that AKI is an inflammatory process involving complex interactions between vascular, tubular, and inflammatory factors. It is often precipitated by sepsis/systemic inflammatory response syndrome, renal hypoperfusion, volume depletion, and nephrotoxicity. In most cases, the aetiology is multifactorial. Patients with pre-existing comorbidities including diabetes, cirrhosis/hepatic failure, congestive heart failure, pre-existing chronic kidney disease (CKD), peripheral vascular disease and advanced age are at particular risk of AKI during an acute illness.

## 4. Diagnosis of AKI 

The symptoms of AKI may be vague, nonspecific, or even absent. The diagnosis of AKI currently depends on detection of reductions in kidney function by conventional surrogate markers, such as serum creatinine and urine output. However, both are known to have significant limitations. Furthermore, the RIFLE, AKIN, and KDIGO criteria for AKI are based on relative changes in serum creatinine from baseline. If a baseline creatinine is not known it would seem prudent to assume that any elevated laboratory creatinine is AKI until proven otherwise. Furthermore, creatinine rises are typically seen 24–36 hours after the initial insult which means that critically ill patients may already have significant AKI even if their serum creatinine has not changed yet. The diagnostic workup of patients with AKI depends on the history, clinical signs, and patient characteristics (Table 2).

## 5. Management of AKI

Early recognition of at-risk patients is paramount followed by timely resuscitation with fluids and/or vasoactive drugs and prevention of further renal insults.

### 5.1. Fluid Therapy

The most commonly used fluids are crystalloids and colloids, including gelatins, human albumin, and hydroxyethyl starches (HES) with varying effects on volume expansion. Ideally, balanced crystalloid solutions should be used, as 0.9% saline is associated with hyperchloremic metabolic acidosis and worsening acid-base balance. Data on the safety of different colloids are conflicting [13]. High molecular weight HESs have been associated with an increased risk of AKI, greater need for RRT, and higher mortality and should therefore be avoided [14–16]. Human albumin has been judged to be safe [14].

Although timely fluid resuscitation is important to prevent AKI in conditions associated with volume depletion, there is increasing evidence that excessive fluid administration can be harmful and lead to dysfunction of other organs and adverse outcomes [17, 18]. To avoid both hypo- and hypervolaemia, patients need to be assessed regularly, and fluid prescriptions should be individualised and tailored to the cardiovascular status of the patient. Ultrasound sonography has emerged as a tool to aid the evaluation of fluid status and response to fluid challenges [19, 20]. However, if in doubt, invasive haemodynamic monitoring may be necessary to distinguish fluid responsive patients from those who are hypotensive due to vasodilation.

### 5.2. Vasoactive Drugs

In healthy conditions, renal blood flow is stable within a wide range of mean arterial pressure (MAP) due to renal autoregulation. However, in critically ill patients, in particular in septic shock, derangements in microcirculation and vasoreactivity tend to increase the MAP threshold that guarantees autoregulation. Data on the optimal MAP to prevent the development and/or progression of AKI are conflicting. Current recommendations for the prevention of AKI in the ICU propose to achieve a MAP ≥65 mmHg but indicate that this target pressure should be individualised when possible, especially in patients with pre-existing chronic hypertension [15]. There is good evidence that Noradrenaline in vasodilatory shock can increase renal blood flow, restore urine output, and improve creatinine clearance [21, 22]. In contrast, there is no role for “renal-dose” dopamine in either preventing or ameliorating AKI in critically ill patients [23, 24].

### 5.3. Diuretics

There is no role for diuretics in preventing or treating established AKI. Three meta-analyses confirmed that the use of diuretics in established AKI did not improve renal function or change mortality but carried a significant risk of side effects, including electrolyte derangement, ototoxicity, and vestibular dysfunction [25–27]. There is also no role for diuretics in speeding up recovery of renal function [28]. However, in patients with progressive fluid accumulation, diuretics can minimize fluid overload and may make patient management easier, especially if RRT is not immediately available.

### 5.4. Other Pharmacological Therapies

There is no evidence for routine use of natriuretic peptide, aprotinin, thyroxine, statins, activated protein C, steroids, or calcium channel blockers to prevent AKI in patients who are critically ill [15, 29, 30].

### 5.5. Discontinuation of Potentially Nephrotoxic Drugs

Together with targeted fluid and haemodynamic resuscitative procedures, it is essential to stop/limit all nephrotoxic drugs. When selected medications are considered vital, doses need to be adjusted regularly based on changes to kidney function.

## 6. Prevention of AKI in Specific Patient Groups

### 6.1. Cardiac Disease

The incidence of AKI in acute decompensated heart failure and acute coronary syndrome is estimated to be 24–45% and 9–19%, respectively [31]. Any treatment for acute heart failure aimed at increasing cardiac output and renal blood flow should also lead to improved renal function. Therapeutic options include fluid therapy, inotropic support and if necessary, cautious introduction of noradrenaline but occasionally more invasive treatments like an intra-aortic balloon pump or left ventricular assist device may be required.

### 6.2. Rhabdomyolysis

Estimates from small studies suggest that 20–50% of patients with rhabdomyolysis develop AKI as a result of intravascular fluid depletion, fluid sequestration in injured muscle, renal hypoperfusion, intratubular heme pigment cast formation, and tubular obstruction. There may be additional secondary renal injury due to free radical production and myoglobin-induced nitric oxide scavenging. The cornerstone for prevention of myoglobinuric AKI consists of aggressive volume resuscitation with isotonic crystalloid solutions at 10–15 mL/kg/hr aiming for a target urine output of >200 mL/hr [32]. Isotonic bicarbonate (NaHCO_3_) at 50–100 mmol/L can be added aiming for a urine pH > 6.5 to increase the solubility and renal excretion of tubular myoglobin. Although there are theoretical benefits for adding mannitol (inhibition of intratubular myoglobin deposition and cast formation, and osmotic diuresis), these benefits are not supported by randomised controlled trials [33].

### 6.3. Contrast Exposure

The risk of developing contrast-induced nephropathy (CIN) is determined by pre-existing renal function, presence of risk factors, and the volume of radiocontrast agent. Patients with advanced age, diabetes, pre-existing CKD, haemodynamic instability, and myeloma are at particular risk. Crystalloid fluids are usually recommended for prevention [15, 34]. Both protocolised administration of 0.9% saline or isotonic NaHCO_3_ have the potential to reduce the incidence of CIN with some evidence that sodium bicarbonate may be superior to saline. The role of N-Acetylcysteine (NAC) is controversial due to inconsistent results observed across multiple studies. Several meta-analyses have reported a risk reduction with NAC but also highlighted significant study heterogeneity and positive reporting bias [35–38]. In addition, concern has been raised that the variable efficacy of intravenous NAC against CIN could be explained by differences in study populations and direct effects of NAC on measured serum creatinine levels. As a result, the working party of the European Society of Intensive Care suggests not to use NAC as prophylaxis against CIN [15]. In contrast, the American Thoracic Society Ad Hoc Committee on Acute Renal Failure recommended that in patients at high risk of CIN, intravenous NAC in combination with fluids may be considered but pointed out that the evidence was not sufficient [34]. Diabetic patients who are taking metformin are at risk of developing lactic acidosis following contrast exposure, especially in case of pre-existing CDK [39]. It seems prudent to discontinue metformin for at least 48 hours after contrast exposure and to restart it only if there are no signs of nephrotoxicity.

## 7. Management of Uraemic Complications

In patients with advanced AKI, renal support should be started before the onset of life-threatening uraemic complications. In fact, many patients develop AKI in the community but may not present to the Emergency Department until they develop uraemic symptoms. Patients with AKI and progressive hyperkalaemia (K^+^ > 6.5 mmol/L), refractory metabolic acidosis, progressive pulmonary oedema, and/or uraemic pericarditis need to be referred to the renal or critical care team urgently. Whilst waiting for RRT, medical treatment may be considered.

### 7.1. Medical Treatment of Hyperkalaemia

Severe hyperkalaemia (serum potassium >6.5 mmol/litre) can cause cardiac arrhythmias and should be treated immediately. Initial management consists of stabilization of the myocardium with calcium gluconate or calcium chloride [40]. Although this is a short-term measure only and will not reduce serum potassium, it reduces the arrhythmic threshold. A recent review by the Cochrane Collaboration confirmed that inhaled beta-agonists, nebulised beta-agonists, and intravenous insulin and glucose were all effective at reducing potassium levels, in particular when administered in combination [40]. The evidence for the use of intravenous NaHCO_3_ to treat hyperkalaemia was considered to be equivocal. Potassium-absorbing resins have no role in the emergency management of hyperkalaemia mainly due to the delay in effect. There is also no clear evidence that means to increase urine output and potassium excretion (e.g., by fluid resuscitation or by loop diuretics) are sufficient to correct hyperkalaemia. If medical therapy fails to correct hyperkalaemia, RRT needs to be considered. 

### 7.2. Medical Management of Fluid Overload/Pulmonary Oedema

If significant respiratory failure is present, this must be dealt with urgently, through supplementary oxygen, noninvasive ventilation, or intubation and ventilation, depending on the state of the patient [41]. While these measures are being undertaken, pharmacological treatment to induce diuresis and offload the decompensated heart can be started, including intravenous opioids, an intravenous infusion of nitrate and diuretics to provoke a diuresis. Converting oliguric to nonoliguric renal failure may help with fluid and electrolyte management, but does not alter the course of AKI and does not change the eventual need for dialysis or overall mortality. Importantly, diuretics may delay the start of RRT otherwise indicated. If medical therapies are not successful, or if the patient is in extremis such that diuretics seem unlikely to prove effective, fluid removal by RRT is the definitive answer.

### 7.3. Medical Management of Metabolic Acidosis

Adverse effects of acute metabolic acidosis primarily include decreased cardiac output, arterial dilatation with hypotension, altered oxygen delivery, decreased ATP production, predisposition to arrhythmias, and impairment of the immune response. In patients with severe metabolic acidosis (pH < 7.1) and evidence of cardiovascular compromise, administration of NaHCO_3_ should be considered to maintain blood pH at ~7.2 [42]. If NaHCO_3_ is administered, it should be given as an isotonic preparation (to prevent hyperosmolality) and as a slow infusion rather than an intravenous bolus (to reduce generation of CO_2_), in quantities designed to raise blood pH to levels not greater than 7.2. Isotonic (1.26%) solutions may also have a role in stable patients with a moderate to severe acidosis and a requirement for fluid replacement, in whom RRT is not imminent.

### 7.4. Management of Uraemic Pericarditis

The prevalence of uraemic pericarditis in patients with acute or chronic kidney disease is 6–10%. Affected patients are often asymptomatic but may be complaining of chest pain. They often have a small to moderate pericardial effusion which can progress to significant haemodynamic compromise. The only therapy is RRT and pericardiocentesis in case of significant pericardial effusions. Therefore, the presence of a pericardial rub on clinical examination and classic ECG changes consistent with pericarditis are indications for urgent RRT and an urgent echocardiogram.

## 8. Future Developments

The characterisation of novel AKI biomarkers has improved the understanding of the pathophysiology of AKI and provided tools to diagnose AKI earlier than serum creatinine. Neutrophil gelatinase-associated lipocalin (NGAL) is one of the most promising new biomarkers and has been extensively studied in different patient populations [43, 44]. In a prospective study of 635 adults presenting to the ED, a single elevated urinary NGAL result was shown to predict the development of AKI, need for RRT and/or need for ICU admission [45]. More research is necessary to determine the role of these new biomarkers in clinical practice.

## 9. Conclusions

ED physicians should consider the possibility of AKI or incipient AKI in all of their critically ill patients and remember that even small rises in serum creatinine are associated with detrimental short- and long-term effects. At-risk patients need to be identified early. Fluid and haemodynamic resuscitation should be prompt, but patients need to be regularly monitored and reviewed to prevent fluid overload. There is no curative therapy for AKI. Secondary prevention with cessation of nephrotoxic medications is essential. More research is necessary to explore whether any other strategies in the ED could improve the prognosis of patients with AKI.

## Figures and Tables

**Table:** (a) RIFLE classification [11]

RIFLE category	SCr/GFR criteria	Urine output criteria
Risk	↑ SCr ≥150–200% (1.5–2 fold) OR decrease of GFR >25%	Urine output <0.5 mL/kg/hour for 6 hours
Injury	↑ SCr >200–300% (>2-3 fold) OR decrease of GFR >50%	Urine output <0.5 mL/kg/hour for 12 hours
Failure	↑ SCr >300% (>3 fold) from baseline *or* decrease of GFR >75% OR serum creatinine ≥4 mg/dL with an acute rise of ≥44 *μ*mol/L	Urine output <0.3 mL/kg/hour for 24 hours or anuria for 12 hours
Loss	Complete loss of renal function for >4 weeks	
End stage kidney disease	Need for RRT for >3 months	

**Table:** (b) AKI Network classification [12]

AKIN stage	Serum creatinine criteria	Urine output criteria
1	↑ SCr ≥26.4 *μ*mol/L in ≤48 hours OR ↑ SCr ≥150–200% (1.5–2 fold) from baseline	<0.5 mL/kg/h for >6 h
2	↑ SCr >200–300% (>2–3 fold) from baseline	<0.5 mL/kg/h for >12 h
3	↑ SCr >300% (>3 fold) from baseline OR SCr ≥354 *μ*mol/L with an acute rise of ≥44 *μ*mol/L OR treatment with RRT	<0.3 mL/kg/h for 24 h OR anuria for 12 h

**Table:** (c) KDIGO classification [3]

Stage	Serum creatinine criteria	Urine output criteria
1	1.5–1.9 times baseline OR ≥0.3 mg/dL (>26.5 *μ*mol/L) in ≤48 hours	<0.5 mL/kg/h for 6–12 hours
2	2–2.9 times baseline	<0.5 mL/kg/h for ≥12 hours
3	≥3 times baseline OR increase in SCr to ≥4.0 mg/dL (353.6 *μ*mol/L) OR initiation of RRT	<0.3 mL/kg/h for ≥24 hours OR anuria for ≥12 hours

Abbreviations: GFR: glomerular filtration rate; RRT: renal replacement therapy; SCr: serum creatinine.

Only one criterion needs to be met to be classified as AKI; if both are present, the criterion which places the patient in the higher stage of AKI is selected.

**Table 2:** Diagnostic work-up of patients with AKI.

Investigations	Comments
*Essential tests *	
Urinalysis	Proteinuria and/or haematuria represent an active urinary sediment suggestive of glomerular disease
Serum creatinine, urea and electrolytes	
Full blood count and blood film	To rule out thrombotic mircoangiopathy and haemolysis; eosinophilia may be present with interstitial nephritis
C-reactive protein	elevated in inflammatory diseases and/or infections
Arterial or venous bicarbonate	
*Investigations to be considered depending on history and/or clinical signs *	
Creatine kinase	To rule out rhabdomyolysis
Serum and urine protein electrophoresis	To rule out myeloma
Antinuclear antibody (ANA)	In case of possible diagnosis of SLE or connective tissue disease
Antineutrophil antibody (ANCA)	In case of possible systemic vasculitis
Anti-streptolysin O titres	To rule out post streptococcal glomerulonephritis
Anti-glomerular basement membrane antibody	To rule out Goodpasture's disease
Complement levels	Reduced in SLE, infectious endocarditis and cryoglobulinaemia
Hep B, Hep C and HIV serology	To rule out renal disease caused by viral infections
Renal ultrasound	To assess renal size; to rule out obstruction and chronic kidney damage
